# Coagulation Profiles Are Associated With Early Clinical Outcomes in Neonatal Encephalopathy

**DOI:** 10.3389/fped.2019.00399

**Published:** 2019-10-01

**Authors:** Deirdre Sweetman, Lynne A. Kelly, Zunera Zareen, Beatrice Nolan, John Murphy, Geraldine Boylan, Veronica Donoghue, Eleanor J. Molloy

**Affiliations:** ^1^Neonatology, National Maternity Hospital, Dublin, Ireland; ^2^Paediatrics, Royal College of Surgeons in Ireland, Dublin, Ireland; ^3^National Children's Research Centre, Dublin, Ireland; ^4^Paediatrics, Children's Health Ireland (CHI) at Tallaght and Trinity Translational Medicine Institute, Trinity College Dublin, St. James Hospital, Dublin, Ireland; ^5^Haematology, CHI at Crumlin, Dublin, Ireland; ^6^Neonatal Brain Research Group, Cork University Maternity Hospital, Cork, Ireland; ^7^Radiology, National Maternity Hospital, Dublin, Ireland; ^8^Neonatology, CHI at Crumlin, Dublin, Ireland; ^9^Neonatology, Coombe Women's and Infant's University Hospital, Dublin, Ireland

**Keywords:** coagulopathy, neonatal encephalopathy, hypoxic-ischaemic encephalopathy, EEG, MRI

## Abstract

**Introduction:** Neonatal encephalopathy (NE) is associated with coagulation abnormalities. We aimed to investigate the serial alterations in coagulation profiles in term infants with NE and correlate with their clinical outcomes. This was a prospective cohort study in a tertiary referral, university-affiliated maternity hospital. Neonates exposed to perinatal asphyxia were recruited (*n* = 82) and 39 received therapeutic hypothermia. Infants had serial coagulation tests including platelets, prothrombin time (PT), activated partial thromboplastin time (aPTT) and fibrinogen in the first week of life. The main outcome measures included MRI brain and EEG seizures. Our results show that mortality was predicted on day 1 by decreased Fibrinogen (AUC = 0.95, *p* = 0.009) and by PT on day 2 with a cutoff of 22 s. An abnormal MRI was predicted by Fibrinogen on day 3 with a cut-off value of 2 g/L. For prediction of grade II/III NE, PT on day 2 of life was strongest with a cut-off value of 14 s. Only elevated APTT levels on day 1 of life were predictive of seizures (AUC = 0.65, *p* = 0.04).

**Conclusion:** Coagulation parameters are strong predictors of outcomes such as abnormal NE grade, seizures, and mortality.

## Introduction

Coagulopathy may occur as part of the spectrum of multi-organ dysfunction following a perinatal hypoxic-ischaemic insult (1). Newborn term infants who have been exposed to an asphyxial insult are at higher risk of disseminated intravascular coagulation (DIC) compared to normal term newborns (2–4). Disturbances in coagulation function are common and manifest with reduced fibrinogen levels, prolonged prothombin time (PT) and activated partial thromboplastin time (APTT) (5, 6). Following a hypoxic, ischaemic, or septic insult a process of consumptive coagulopathy occurs with raised levels of fibrin degradation products (FDPs), prolongation of the PT and decreased platelet counts. A hypocoagulable state is also thought to develop with prolongation of the APTT (7). Plasma levels of thrombin-anti-thrombin complexes, D-dimers, fibrin degradation products, and soluble fibrin monomer complexes are also reported to be higher in the asphyxiated term infant correlating with disseminated intravascular coagulation (2–4). Compared to dysfunction of other organs such as the heart or liver, in neonatal encephalopthy (NE) there are few published studies that have looked prospectively at the abnormalities in coagulation and hemostasis and correlated them with outcome or severity of NE (8). Forman et al. found that infants with NE (*n* = 76) had bleeding events in 54% of cases but that all infants had coagulopathy (9). Therapeutic hypothermia in both children and adults is associated with coagulopathy although not sufficiently severe to require discontinuation of TH (10).

We hypothesized that there is an association between term babies with neonatal encephalopathy who are at risk of coagulation abnormalities, and severity of NE and outcome. We aimed to confirm that coagulation abnormalities observed in neonates with NE, may be used in the prediction of adverse neonatal outcomes, represented by the severity of neonatal encephalopathy, neonatal mortality, seizures development, as well as altered MRI.

## Methods

The study was approved by the Ethics Committee of the National Maternity Hospital, Dublin, a tertiary referral, University-affiliated Maternity hospital with ~9,500 deliveries per annum for the study period and is one of four national referral centers for Therapeutic Hypothermia. In all cases, written informed consent was taken from parents of infants enrolled in the study for the research period of February 2011–December 2012. The following infants were enrolled: neonates exposed to perinatal asphyxia (PA) as previously described (11). Infants with congenital abnormalities or evidence of maternal substance abuse were excluded. On completion of the study, infants exposed to PA were divided into subgroups according to the classification of Sarnat and Sarnat (12): (a) NE 0/I: infants who required resuscitation following delivery with no neurological signs or mild encephalopathy; (b) NE II/ III: moderate/severe encephalopathy.

### Coagulation Screens

Serial coagulation samples were taken as part of routine care. Arterial samples were taken when peripheral or umbilical arterial catheters were *in situ*, otherwise peripheral venous samples were obtained including precautions to prevent heparin sample contamination (13). Coagulation screens were analyzed using the Sysmex CS-2100i coagulation analyser and full blood counts as part of validated laboratory practices.

### Data and Statistical Analysis

**Figure 1:**
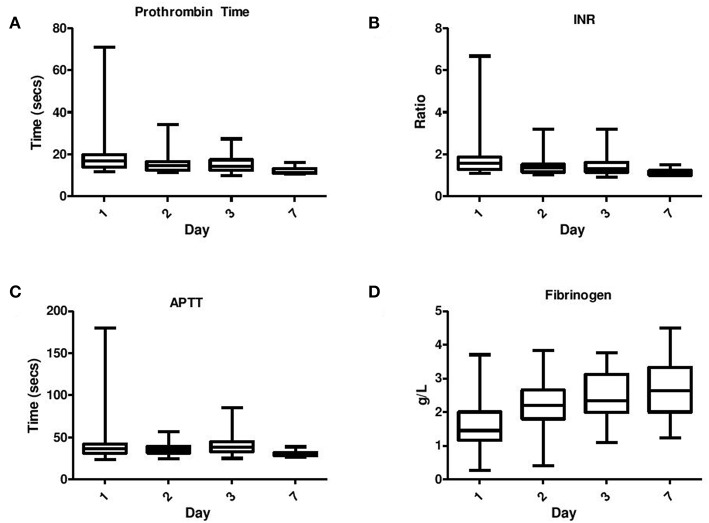
Serial daily coagulation values in neonatal encephalopathy analyzed on day 1–day 7 including **(A)** Prothrombin time, **(B)** INR, **(C)** APPT, and **(D)** Fibrinogen.

Coagulation results were available from 79 of the 82 infants enrolled in the study. If more than one coagulation screen was taken each day, the maximum value for PT, APTT, and INR was recorded for each time point while the minimum value for fibrinogen and platelets was recorded for each time point. In general the data showed a skewed distribution and therefore medians, interquartile ranges, minimums, and maximums were calculated (Figure 1). Non-parametric tests i.e., the Mann–Whitney *U*-test was utilized to compare each coagulation parameter at each time point with the treatment Therapeutic Hypothermia and outcomes such as seizure occurrence, grouping of grade of NE, MRI brain result, and mortality.

Maximum PT, APTT, and minimum fibrinogen levels were compared to therapeutic hypothermia and outcomes as previously listed using the Mann–Whitney *U*-test (**Table 2**). For certain comparisons i.e., those with a number of <5 observations in one of the groups, it was necessary to run a Kruskal–Wallis test with Monte Carlo significance to analyse this data more closely. Receiver Operating Characteristic Curve analysis was employed to assess the coagulation parameters for ability to predict outcome. Using the student-*t*-test, the means of all the coagulation variables were compared to TH and to outcome measures: occurrence of seizures, grouping of grade of NE and MRI brain result.

Statistical analysis was carried out using the PASW statistical package version 18 (www.ibm.com/SPSS_Statistics). Normally distributed data were expressed using means ± standard deviations (SD) and the Student *t*-test, while skewed data were expressed using medians and IQRs and the Mann– initially assumed for values of *p* < 0.05 with 95% confidence interval (CI). A Bonferroni correction for multiple testing was employed by altering the threshold for “significance.” This was achieved by dividing the threshold for “significance” that is 0.05 by the number of tests carried out. We left *in situ* the original *p*-values but annotated the *p*-values which still retained significance at a Bonferroni corrected level. Receiver operating characteristic (ROC) curve analysis was employed amongst the PA infants looking at the outcome of NE grade and cut-off values were calculated.

## Results

The study included infants exposed to PA (*n* = 82) and the mean values (SD) were as follows within NE 0/I vs. NE II/III: gestation: 39.9 ± 1.6 vs. 40.6 ± 1.6 weeks; birth weight: 3.5 ± 0.7 vs. 3.7 ± 0.7 kg and male gender: 69 vs. 64%. Thirty-nine infants required TH in accordance with the TOBY (Total Body Hypothermia for Neonatal Encephalopathy) criteria (13) and were treated for 72 h duration. Clinical seizures developed in 39 infants and 4 infants died. The NE group II/III were significantly more likely to have the following features: outborn, lower 1, 5, and 10 min Apgar scores, clinical seizures, TH, abnormal MRI brain imaging, and significantly lower admission pH, admission bicarbonate, higher ALT, AST, and CK levels and larger base excess values compared to NE group 0/I. However, there were no significant differences between the NE groups 0/I and II/III with regard to gestational age, birth weight, gender, mode of delivery, multiplicity, mortality, cord pH, cord base excess, and cord lactate values as previously described (11).

MRI brain scans were performed on 66 infants (normal *n* = 35) and scored and reported independently by a pediatric radiologist according to the Barkovich criteria divided into “normal” (score = 0) and “abnormal” (score = 1–4) neuroimaging groups for the purpose of data analysis (11). Barkovich scores were available for the MRI brain scans of 63 infants with NE giving the following results: Basal Ganglia (BG) score: 1 (*n* = 3); 2 (*n* = 2); 3 (*n* = 2); 4 (*n* = 6). Watershed (W) score = 1 (*n* = 7); 2 (*n* = 3); 4 (*n* = 3); 5 (*n* = 3). Combined Basal Ganglia/Watershed (BG/W) score = 1 (*n* = 5); 2 (*n* = 10); 3 (*n* = 6); 4 (*n* = 1).

### Coagulation Results

Eighty-two infants were recruited to the study and five infants showed evidence of frank bleeding in at least one of the following categories: pulmonary hemorrhage (*n* = 3); frank hematuria (*n* = 3); significant umbilical bleeding (*n* = 2); nasal bleed (*n* = 1); fresh blood in gastric aspirates (*n* = 3). Four infants demonstrated evidence of minor bleeding: leakage of umbilical serosanguinous fluid (*n* = 3); post capillary blood sampling bleeding (*n* = 1). Excluding the finding of a cephalohaematoma, only one infant had significant superficial bruising. Another infant was later diagnosed aged 16 months with a mild thrombophilia disorder (Factor V Leiden deficiency). Platelet levels were significantly lower in infants with frank bleeding compared to the remaining infants (mean ± SD: 79 ± 45 × 10^9^/L vs. 170 ± 70 × 10^9^/L; *p* = 0.001). In the infants with frank bleeding (pulmonary, hematuria, umbilical, nasal, or gastric) the coagulation results were as follows: the average maximum PT was 52 s, Max aPTT was 94 s and Minimum Fibrinogen 0.64 s.

Twenty-six infants required at least one blood product during their NICU admission (Table 1). In total there were 94 blood products administered to the infants with NE over the study period including additional vitamin K. Vitamin K was the most commonly administered medication for correction of prolonged PT (*n* = 28) with red cell transfusion being the most common form of blood product given (*n* = 18 transfusions). Human Pooled Plasma, Solvent/Detergent Treated (Octaplas *n* = 16) was much more commonly administered than Fresh Frozen Plasma (FFP *n* = 2).

**Table 1:** Blood products requirements in infants with NE.

**Blood product**	No. of infants^*^	**No. of blood products^π^**
Red cell transfusion	10	18
Platelets	8	12
Octaplas	10	16
Fresh frozen plasma	2	2
Fibrinogen	9	12
Vitamin K	16	28
Albumin 20%	5	6
Total	60	94

* *Number of Infants receiving at least one dose of each blood product*.

π *Total numbers of each type of blood product received by study infants*.

The Chi-squared test was used to compare the categorical variables of TH, presence of seizures, grade of encephalopathy by group, MRI brain result and the hematological categorical variables of frank bleeding, pulmonary hemorrhage, minor bleeding, bruising/petechiae, thrombophilia, and requirement for blood products. There was a significant association between grouping of grade of NE i.e., normal (Sarnat grade 0 and I) and abnormal (Sarnat grade II and III) and requirement for blood products [$\chi_{\left(1\right)}^{2}$ 10.88, *p* = 0.001, OR 7.45]. An infant with NE II/III was 7.45 times more likely to require blood products. There was a significant association between TH and requirement for blood products [$\chi_{\left(1\right)}^{2}$ = 11.16, *p* = 0.001, OR = 5.31]. If an infant required TH, they were 5.31 times more likely to require blood products than if they were not cooled. The occurrence of seizures was not associated with any of the hematological variables measured, including requirement for blood products. There was no significant association between MRI result or neurological examination at discharge and any of the clinical hematological variables measured (Table 2).

**Table 2:** Relationship between hematological and short-term outcome variables in NE.

**Hematological variables**	**TH**		**Seizures**		**NE group**		**MRI**	
	**() χ^2^**	***p* (π)**	**() χ^2^**	***p* (π)**	**() χ^2^**	***p* (π)**	**() χ^2^**	***p* (π)**
Frank bleeding	(1) 6.27	0.01^*^	(1) 0.43	0.51^*^	(1) 3.21	0.07^*^	(1) 0.56	0.45^*^
Pulm haem	(1) 3.67	0.06^*^	(1) 3.67	0.06^*^	(1) 1.88	0.17^*^	(1) 2.46	0.12^*^
Minor bleeding	(1) 4.95	0.03^*^	(1) 4.95	0.03^*^	(1) 2.53	0.11^*^	(1) 1.48	0.22^*^
Bruise/petechia	(1) 1.19	0.28^*^	(1) 1.19	0.28^*^	(1) 0.61	0.43^*^	(1) 0.85	0.36^*^
Thrombophilia	(1) 0.86	0.35^*^	(1) 1.19	0.28^*^	(1) 0.61	0.43^*^	(1) 1.21	0.27^*^
Blood product requirement	(1) 11.16	0.001 (5.31)	(1) 2.1	0.15	(1) 10.88	0.001 (7.45)	(1) 1.05	0.31

χ, Chi-squared test employed for comparisons; χ^2^, Chi-squared test statistic (degrees of freedom =); p, p < 0.05 taken as significant;

* *Expected count less than 5, therefore the chi-square test accuracy unreliable; π, Odds ratio if significant; Pulm Haem, Pulmonary Hemorrhage; TH, Therapeutic Hypothermia*.

PT, INR, and APTT levels on day 1–3 were significantly elevated in infants undergoing TH (*p* ≤ 0.001). While fibrinogen levels were significantly decreased in infants who received TH only on day 1 and 2 of life (*p* = 0.001, 0.007, respectively). The APTT level on day 1 of life was the only coagulation parameter significantly associated with seizure occurrence (*p* = 0.04). PT, INR, and APTT levels on day 1, 2, and 3 were significantly elevated in infants with grade II/III NE (all *p* < 0.002). While only fibrinogen levels on day 1 were significantly decreased in infants with grade II/III NE (*p* = 0.04). PT and INR levels on day 7 of life were significantly elevated in infants with an abnormal MRI result (*p* = 0.03) and fibrinogen levels on day 3 were significantly decreased (*p* = 0.02). Infants who died had significantly higher PT, INR, and APTT levels on day 1 (*p* = 0.007, 0.006, 0.007) and day 2 (*p* = 0.006, 0.008, and 0.04), respectively. However, death was significantly associated with lower fibrinogen levels only on day 1 of life (*p* = 0.009) (Figure 1).

Maximum PT values for each patient were significantly elevated in infants who underwent TH, had grade II/III NE or died (*p* < 0.001, *p* < 0.001, *p* = 0.002, respectively). Maximum APTT values were significantly elevated in infants who underwent TH, had seizures, NE II/III, and those who died (*p* < 0.001, *p* = 0.048, *p* < 0.001, and *p* = 0.003, respectively). Minimum fibrinogen values for each patient were significantly lower in those infants who underwent TH, had grade II/III NE and those who did not survive (*p* < 0.001, *p* = 0.03, and *p* = 0.002, respectively) (Table 3). Grade II/III NE was significantly associated with lower mean (SD) platelet count on day 2, 3, and 6 [157.5(65.5) vs. 220.1(54.7); 154.9(75.2) vs. 235.3(73.3); 136.4(85.1) vs. 299.5(62.9) × 10^9^/L, respectively]. Platelet counts were significantly negatively associated with abnormal NE II/III on day 1, 2, 3, and 6 (*p* = 0.02, 0.001, 0.006, and 0.02, respectively). There were no significant associations found between maximum PT, APTT, and minimum fibrinogen and infants with an abnormal MRI brain result (Table 3).

**Table 3:** Associations between TH, outcome measures and coagulation parameters of infants with NE.

**Haem variable**		**TH**		**NE Grade**		**Death**	
	**D**	**Median**	***p***	**Median**	***p***	**Median**	***p***
PT	1	17.5 (16.1–22.7), 13.9 (13–17.1)	<0.001	17.8 (16–22.7), 13.9 (13.2–16.6)	<0.001	53.9 (22.9–53.9)^*^, 16.7 (14–19.3)	0.007
	2	15.8 (14.3–20.3), 12.5 (11.4–13.4)	<0.001	15.3 (13.9–19.6), 12.5 (11.5–13.4)	<0.001	32.8 (22.6–32.8)^*^, 14.4 (12.5–16.1)	0.006
	3	16.1 (13.6–17.9), 10.9 (10.5–13.5)	<0.001	14.7 (13.4–17.6), 10.6 (10.1–10.9)	0.002	21.7 (15.9–21.7)^*^, 13.9 (12.4–17.4)	0.15
	7	N/A	N/A	11.5 (10.9–13.3), 11.4^*^	0.71	N/A	N/A
APTT	1	40.3 (34.8–46.6), 31.3 (27.3–35.6)	<0.001	39.4 (33.2–45.2), 31.4 (28.7–35.6)	<0.001	71.1 (45.6–71.1)^*^, 35.8 (31.2–40.7)	0.008
	2	37.8 (35.2–45.3), 30.9 (27.8–33.9)	<0.001	36 (33.2–42.6), 31 (28–34)	0.002	42.8 (41.9–42.8)^*^, 34.5 (31.1–38.1)	0.04
	3	41.7 (38–48.6), 29.1 (26.2–33.5)	<0.001	39.1 (34.4–45.8), 26.2 (25.1–28)	0.002	45.9 (43.9–45.9)^*^, 38 (32.2–44.3)	0.18
	7	N/A	N/A	30.1 (28.3–32), 31.5^*^	0.39	N/A	N/A
Fib	1	1.3 (1–1.7), 1.8 (1.4–2.1)	0.001	1.3 (1–2), 1.7 (1.4–2.1)	0.04	0.61 (0.27–0.61)^*^, 1.5 (1.2–2)	0.009
	2	2.1 (1.7–2.4), 2.5 (2.2–3.1)	0.007	2.1 (1.7–2.6), 2.5 (2.1–3.1)	0.05	1.9 (0.4–1.9)^*^, 2.2 (1.8–2.7)	0.07
	3	2.1 (2–3), 2.6 (1.8–3.2)	0.59	2.2 (1.9–3), 2.8 (2.4–3.6)	0.15	2.0 (2–2)^*^, 2.4 (2.0–3.1)	0.23
	7	N/A	N/A	2.6 (2–3.3), 2.8^*^	0.71	N/A	N/A

Medians (IQR) of cooled vs. non-cooled infants, abnormal vs. normal NE grade and death vs. survival, followed by a p-value (p); D, Day; PT, Prothrombin Time (secs); APTT, Activated Partial Thromboplastin Time (secs); Fib, Fibrinogen (g/L); N/S, Not significant; N/A, Not applicable due to missing values;

* *No IQR available due to small numbers*.

Day 3 values for PT, APTT and fibrinogen when compared to NE grade fitted this criteria. Using Kruskal-Wallis, the significant association between elevated PT, APTT, and grade II/III NE remained (mean rank 21.43 vs. 3.13, Monte Carlo *p* < 0.001 and mean rank 20.91 vs. 3.25, Monte Carlo *p* < 0.001, respectively). With regard to the outcome of mortality there were small numbers in one of the groups for PT, APTT, and fibrinogen values across day 1–3. Further analysis of these comparisons using the Kruskal–Wallis test and Monte Carlo significance revealed that the significant associations between higher PT and APTT values on day 1 and 2 and lower fibrinogen values on day 1 in those infants who died, remained true. Similarly for maximum PT, APTT, and minimum fibrinogen values for each patient there remained a significant association between higher maximum PT, APTT values, and lower minimum fibrinogen values with mortality (Table 4).

**Table 4:** Comparison of maximum PT, APTT, and minimum fibrinogen values for each infant with outcomes.

**Coag value**	**TH**		**Seizures**		**NE grade**		**Death**	
	**Rank**	***p***	**rank**	***p***	**Rank**	***p***	**Rank**	***p***
Max PT	54.37, 25.99	<0.001	43.33, 36.75	0.20	25.37, 47.18	<0.001	74.0, 38.19	0.002
Max APTT	56.83, 23.59	<0.001	45.18, 34.95	0.048	22.96, 48.36	<0.001	73.25, 38.23	0.003
Min fib	27.44, 52.25	<0.001	39.73, 40.26	0.92	48.25, 35.95	0.03	5.5, 41.84	0.002

**Figure 2:**
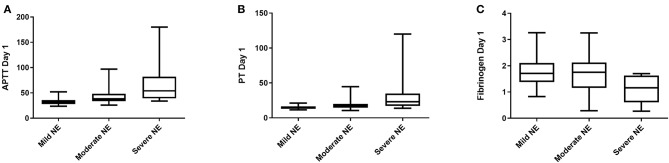
Coagulation screens and correlation with clincial outcomes. **(A)** APTT, Activated Partial Thromboplastin Time (s); **(B)** PT, Prothrombin Time (s); **(C)** Fib, Fibrinogen (g/L). Grade I NE, Mild; Grade II NE, Moderate; Grade III NE, Severe.

In order to look more closely at the relationship between coagulation values and grade of encephalopathy (normal = 0 was excluded from analysis, mild = I, moderate = II, severe = III), PT and APTT levels on day 1, 2, 3 and fibrinogen levels on day 1 only, were significantly associated with grade of NE (Figure 2). However, it was not possible to examine this relationship further using an ANOVA as the variance of the data was too different and the data was not normally distributed. Elevated PT and APTT on day 1, 2, and 3 were predictive of TH (*p* < 0.001), while decreased fibrinogen levels on day 1 and 2 were predictive of TH (AUC = 0.75, 0.72, *p* = 0.001, 0.007, respectively). Only elevated APTT levels on day 1 of life were predictive of seizure occurrence (AUC = 0.65, *p* = 0.04). Increased PT and APTT levels on day 1, 2, and 3 of life were predictive of grade II/III NE with excellent AUCs of ≥0.80 (all *p* < 0.002). Low fibrinogen levels on day 1 of life were predictive of abnormal grade II/III NE (AUC = 0.66, *p* = 0.04). Only a raised PT level on day 7 of life along with decreased fibrinogen level on 3 of life were predictive of an abnormal MRI brain scan (AUC = 0.88, *p* = 0.03, AUC = 0.73, *p* = 0.02). Mortality was strongly predicted by raised PT levels on day 1 and 2 of life (AUC = 0.96, *p* = 0.008, AUC = 0.98, *p* = 0.006) and by raised APTT levels on day 1 and 2 of life also (AUC = 0.95, *p* = 0.008, AUC = 0.85, *p* = 0.04). While only decreased fibrinogen levels on day 1 of life were strongly predictive of mortality (AUC = 0.95, *p* = 0.009).

APTT on day 1 of life was the best predictor of seizure occurrence with a cut-off value of 37 s. Fibrinogen on day 3 of life was the best predictor of an abnormal MRI brain scan result with a cut-off value of 2 g/L. Finally for prediction of death, PT on day 2 of life was the best predictor with a cut off value of 22 s to predict mortality.

## Discussion

PT and APTT levels are increased while fibrinogen and platelet levels were significantly decreased in infants who required TH, had grade II/III NE and in non-survivors. Coagulation parameters were strong predictors of outcomes such as abnormal NE grade and death. Cut-off values of PT, APTT, and fibrinogen for predicting such outcomes may in the future be incorporated into a biomarker panel for outcome prediction.

The prothrombin time measures factors I, II, V, VII, X which are all manufactured in the liver and therefore the PT is also a useful test of liver function, since there is a good correlation between abnormalities in coagulation measured by the prothrombin time and the degree of liver dysfunction. The PT measures the extrinsic pathway of coagulation, however in adults, the PT is compared to a normal control sample and given as a ratio called the international normalized ratio (INR) which allows that all results are standardized irrespective of which analytical system is used to measure the PT. In newborns, the actual PT is quoted and the INR is less commonly used (6).

The INR of a group of 26 asphyxiated infants rose significantly on day 1 and 2 of life compared to normal control infants, however was shown to normalize by day 3. Furthermore, Beshlawy et al. showed a marked decrease in the level of the physiological inhibition system of coagulation with significant decreases in mean levels of antithrombin III (38.4 vs. 78.5%), protein C (10.46 vs. 32.63%), and protein S (18.66 vs. 48.66%) in all 10 neonates with NE compared to control infants before the occurrence of thromboembolic complications (5). The following occurred: DIC and died (*n* = 5); necrotizing enterocolitis and rectal bleeding (*n* = 4); hematuria (*n* = 2); hematemesis (*n* = 3); intracranial hemorrhage (*n* = 2); seizures (*n* = 10) and all 10 neonates with NE had prolongation of the PT and APTT. The study concluded that based on the development of antithrombin III and protein C concentrates, which are commercially available, require minimal monitoring, and have very few side effects, there is a strong argument for the prompt evaluation of the optimal treatment for thromboembolic accidents in NE. Pakvasa et al. demonstrated that 69% of infants has hemostatic dysfunction. From a cohort of 98 infants with NE, they noted that 57% required at least one blood product which was predominantly fresh frozen plasma (14). There is controversy regarding transfusion thresholds in neonates and a more conservative approach has been suggested which is more in keeping with the transfusion policy in our study (15, 16). The limitations in this study include the sample size and that timing of sampling was linked with clinical indication and so varied in the first day of life.

Infants with NE have multiorgan dysfunction and coagulopathy is common. In the first few days of life coagulation is measured routinely and this study demonstrates the utility of these routine markers in predicting outcome. Further research to determine cutoff values for interventions such as blood product administration are required.

## What Is Known

Coagulopathy is associated with hypoxia-ischaemia and is described in Neonatal Encephalopathy.

## What Is New

Coagulation markers are associated with seizures, MRI and mortality in Neonatal Encephalopathy.

## Data Availability Statement

The datasets for this study will not be made publicly available because it is confidential patient data.

## Ethics Statement

This study was carried out in accordance with the recommendations of the Ethics Committee of the National Maternity Hospital, Dublin, Ireland with written informed consent from parents of infants enrolled in the study. All subjects gave written informed consent in accordance with the Declaration of Helsinki. The protocol was approved by the Ethics Committee of the National Maternity Hospital, Dublin.

## Author Contributions

DS, LK, ZZ, and EM: drafting the work or revising it critically for important intellectual content. EM: agreement to be accountable for all aspects of the work in ensuring that questions related to the accuracy or integrity of any part of the work are appropriately investigated and resolved. All authors: substantial contributions to the conception or design of the work or the acquisition, analysis or interpretation of data, and final approval of the version published.

### Conflict of Interest

The authors declare that the research was conducted in the absence of any commercial or financial relationships that could be construed as a potential conflict of interest.

## Abbreviations

NE: Neonatal encephalopathy
PT: Prothrombin time
aPTT: Activated partial thromboplastin time
INR: International normalized ratio
DIC: Disseminated intravascular coagulation
PA: Perinatal asphyxia
TH: Therapeutic Hypothermia
ROC: Receiver operating characteristic
TOBY: Total Body Hypothermia for Neonatal Encephalopathy.
